# EGFR-Targeted TRAIL and a Smac Mimetic Synergize to Overcome Apoptosis Resistance in *KRAS* Mutant Colorectal Cancer Cells

**DOI:** 10.1371/journal.pone.0107165

**Published:** 2014-09-08

**Authors:** Yvonne Möller, Martin Siegemund, Sven Beyes, Ricarda Herr, Daniele Lecis, Domenico Delia, Roland Kontermann, Tilman Brummer, Klaus Pfizenmaier, Monilola A. Olayioye

**Affiliations:** 1 Institute of Cell Biology and Immunology, University of Stuttgart, Stuttgart, Germany; 2 Institute of Molecular Medicine and Cell Research (IMMZ), Faculty of Medicine, Albert-Ludwigs-University of Freiburg, Freiburg, Germany; 3 Faculty of Biology, Albert-Ludwigs-University of Freiburg, Freiburg, Germany; 4 Centre for Biological Signalling Studies BIOSS, Albert-Ludwigs-University of Freiburg, Freiburg, Germany; 5 Department of Experimental Oncology, Fondazione IRCCS Istituto Nazionale dei Tumori, Milan, Italy; University General Hospital of Heraklion and Laboratory of Tumor Cell Biology, School of Medicine, University of Crete, Greece

## Abstract

TRAIL is a death receptor ligand that induces cell death preferentially in tumor cells. Recombinant soluble TRAIL, however, performs poorly as an anti-cancer therapeutic because oligomerization is required for potent biological activity. We previously generated a diabody format of tumor-targeted TRAIL termed Db_αEGFR_-scTRAIL, comprising single-stranded TRAIL molecules (scTRAIL) and the variable domains of a humanized variant of the EGFR blocking antibody Cetuximab. Here we define the bioactivity of Db_αEGFR_-scTRAIL with regard to both EGFR inhibition and TRAIL receptor activation in 3D cultures of Caco-2 colorectal cancer cells, which express wild-type K-Ras. Compared with conventional 2D cultures, Caco-2 cells displayed strongly enhanced sensitivity toward Db_αEGFR_-scTRAIL in these 3D cultures. We show that the antibody moiety of Db_αEGFR_-scTRAIL not only efficiently competed with ligand-induced EGFR function, but also determined the apoptotic response by specifically directing Db_αEGFR_-scTRAIL to EGFR-positive cells. To address how aberrantly activated K-Ras, which leads to Cetuximab resistance, affects Db_αEGFR_-scTRAIL sensitivity, we generated stable Caco-2tet cells inducibly expressing oncogenic K-Ras^G12V^. In the presence of doxycycline, these cells showed increased resistance to Db_αEGFR_-scTRAIL, associated with the elevated expression of the anti-apoptotic proteins cIAP2, Bcl-xL and Flip_S_. Co-treatment of cells with the Smac mimetic SM83 restored the Db_αEGFR_-scTRAIL-induced apoptotic response. Importantly, this synergy between Db_αEGFR_-scTRAIL and SM83 also translated to 3D cultures of oncogenic K-Ras expressing HCT-116 and LoVo colorectal cancer cells. Our findings thus support the notion that Db_αEGFR_-scTRAIL therapy in combination with apoptosis-sensitizing agents may be promising for the treatment of EGFR-positive colorectal cancers, independently of their *KRAS* status.

## Introduction

Colorectal cancer (CRC) is one of the most prevalent cancers worldwide and especially in patients with advanced CRC survival rates are low [1]. In addition to chemotherapy, targeted therapies have entered the clinic. Currently, the EGFR (epidermal growth factor receptor) blocking antibodies Cetuximab and Panitumumab are approved for the treatment of metastatic CRC in combination with chemotherapy or as a maintenance therapy in chemo-refractory tumors [2], [3].

EGFR, also known as ErbB1 or HER1, is associated with the pathogenesis of various human epithelial cancers. This receptor tyrosine kinase comprises an extracellular ligand-binding domain, a single membrane spanning region, and a cytoplasmic tyrosine kinase domain [4], [5]. Upon binding of ligands such as EGF and TGF-α, the receptor homo- and heterodimerizes preferentially with the family member ErbB2/HER2 leading to receptor activation and transphosphorylation of specific tyrosines within the cytoplasmic tails. These phosphotyrosines provide docking sites for intracellular signaling molecules that trigger the activation of MAPK and PI3K pathways, which mediate biological responses such as proliferation, migration and survival [5], [6]. Cetuximab competes with EGFR ligands for receptor binding, thereby repressing receptor phosphorylation and the activation of downstream signaling [1].

The different genetic alterations found in CRC limit the efficacy of anti-EGFR therapies. Nearly 40% of all CRC cases harbor activating mutations in the *KRAS* gene. Receptor tyrosine kinase signaling converges at the level of the small GTPase Ras, a master regulator of both, MAPK and PI3K pathways. The most frequent mutations occur at codon 12 or 13, leading to constitutive Ras activation and, consequently, reduced or no response to Cetuximab treatment [7], [8].

TRAIL (tumor necrosis factor-related apoptosis-inducing ligand) is a death ligand that induces apoptosis preferentially in tumor cells via the death receptors TRAILR1 and TRAILR2, also known as DR4 and DR5, respectively [9]. Binding of TRAIL triggers receptor oligomerization, followed by the recruitment of adaptor proteins and the formation of the death-inducing signaling complex. This ultimately leads to the activation of initiator caspases and consecutive activation of effector caspases, resulting in apoptotic cell death [10]. Clinical trials using recombinant TRAIL confirmed the low toxicity to normal tissue, but therapeutic effects were insufficient [11], [12]. To overcome these limitations protein engineering approaches have aimed at improving bioactivity while maintaining tumor selectivity. Correct trimerization and zinc coordination of recombinant TRAIL seem to be crucial for biological activity [13]. Accordingly, the design of a single polypeptide chain comprising the extracellular domains of three TRAIL monomers (scTRAIL) enhanced the bioactivity of the recombinant molecule [14]. Such molecules can further be fused to antibodies directed against tumor markers. We previously showed that the fusion of scTRAIL to a single-chain antibody fragment (scFv) functionally mimicked natural membrane-bound TRAIL and was more effective than scTRAIL alone [14]. The introduction of a diabody configuration based on the humanized variable regions of Cetuximab (Db_αEGFR_-scTRAIL) resulted in an even higher bioactivity of recombinant TRAIL both in vitro and in vivo, as seen by the strong reduction of tumor size and prolonged survival of nude mice carrying Colo205 xenografts [15].

Apart from its tumor targeting effect, the EGFR-directed antibody moiety contained within the Db_αEGFR_-scTRAIL molecule may actively interfere with EGFR function while simultaneously stimulating apoptosis. To dissect the contribution of EGFR blockade to the bioactivity of Db_αEGFR_-scTRAIL we used the EGFR-positive Caco-2 CRC cell line, which harbors mutations in APC, p53, and SMAD4 but is wild-type for the MAPK and PI3K pathways [16]. To mimic more closely the in vivo situation, Caco-2 cells were grown in 3D collagen/matrigel cultures where they form fully differentiated polarized cysts [17]. Growth conditions are known to influence the balance of survival and apoptosis signals, highlighting the need for studying drug treatment and resistance mechanisms not only in conventional 2D cultures [18]. Indeed, our results show that cultivation of Caco-2 cells in a 3D matrix renders cells TRAIL-sensitive. We further demonstrate that EGFR signaling contributes to Caco-2 cell proliferation and can be blocked by pharmacological EGFR inhibition. The importance of the EGFR-specific antibody moiety for the efficient targeting of Db_αEGFR_-scTRAIL is underscored by the fact that low EGFR levels characterize the cell subpopulation that survives Db_αEGFR_-scTRAIL treatment. Moreover, although insensitive to EGFR blockade per se, EGFR-positive Ras mutant CRC cells are targeted and sensitized to Db_αEGFR_-scTRAIL-induced apoptosis by co-treatment with the Smac mimetic SM83. The potent cytotoxic activity of Db_αEGFR_-scTRAIL revealed in this study thus lends support for its further development as an anti-cancer therapeutic for the treatment of CRC.

## Materials and Methods

### Antibodies and reagents

Antibodies used were monoclonal rabbit anti-pEGFR (Y1068) (1∶1000), monoclonal rabbit anti-caspase-3 (1∶1000), polyclonal rabbit anti-TRAILR2 (1∶500), polyclonal rabbit anti-pERK (T202/Y204) (1∶1000), monoclonal rabbit anti-pAKT (T308) (1∶1000), polyclonal rabbit anti-cIAP1 (1∶1000), monoclonal rabbit anti-cIAP2 (1∶1000), monoclonal mouse anti-ERK (1∶1000), monoclonal mouse anti-AKT (pan) (1∶1000), monoclonal mouse anti E-Cadherin (1∶250), monoclonal mouse anti-Smac (1∶1000), polyclonal rabbit anti-Bcl-2 (1∶1000), monoclonal rabbit anti-Bcl-xL (1∶400) and monoclonal rabbit anti-survivin (1∶1000) (all from Cell Signaling, Danvers, MA, USA), monoclonal mouse anti-xIAP (1∶400), monoclonal mouse anti-Ras (1∶200) (BD, CA, SanJose, USA), monoclonal mouse anti-FlipS/L (1∶400) and polyclonal rabbit anti-TRAILR1 (1∶1000) (Santa Cruz Biotechnology, Dallas, TX, USA), monoclonal mouse anti-EGFR (1∶500) (Thermo Scientific, Fremont, MA, USA), monoclonal mouse anti-alpha-tubulin (1∶5000) (Sigma-Aldrich, St Louis, MO, USA), and monoclonal mouse anti-GFP (1∶1000) (Roche Applied Science, Mannheim, Germany). HRP-labeled secondary anti-mouse and anti-rabbit IgG antibodies (1∶10000) were from GE Healthcare (Buckinghamshire, UK). Alexa Fluor 488- and 546-labeled secondary anti-mouse and anti-rabbit IgG antibodies (1∶500) and Alexa Fluor 633-labeled phalloidin (1∶100) were from Invitrogen (Carlsbad, CA, USA). DAPI was from Sigma-Aldrich, Z-VAD-FMK was from Bachem AG (Bubendorf, Switzerland), and Cetuximab from Merck (Darmstadt, Germany). Db_αEGFR_-scTRAIL was produced in HEK293 cells and purified from cell culture supernatants [15]. The synthesis and purification of SM83 was described previously [19], [20].

### Cell culture

Caco-2, HCT-116, and LoVo cell lines were cultured in RPMI 1640 (Invitrogen), and Caco-2tet cells in DMEM (Invitrogen) supplemented with 10% FCS (PAA Laboratories, Cölbe, Germany). Cell lines were incubated in a humidified atmosphere of 5% CO_2_ at 37°C. For growth factor dependent assays cells were cultured in medium containing 2% FCS plus 10 ng/ml EGF (Sigma-Aldrich) and TGF-α (Peprotech, Rocky Hill, NJ, USA). For growth in 3D, cells were seeded on a bed of growth factor reduced matrigel (BD) and PureCol-S collagen (Advanced Biomatrix, San Diego, CA, USA) (1∶1) and overlayed with growth medium containing 2% matrigel. Lumen expansion was induced by addition of 100 ng/ml choleratoxin (CTX; Sigma Aldrich) at day 3 post seeding.

### Generation of Caco-2tet Ras^G12V^ cells

The pTET/*KRAS* ^G12V^-IRES-GFP-bsr expression vector, which allows the doxycycline-inducible expression of K-Ras^G12V^, was generated by recovering the K-Ras^G12V^ open reading frame from pBABE-K-Ras^G12V^ (Addgene) by *BamH*I digestion, followed by the insertion into the *Bgl*II-linearized pMIG vector [21]. The resulting K-Ras^G12V^-IRES-GFP cassette was amplified by PCR using oligonucleotides containing flanking *Not*I sites, which were used for subcloning into pSC-A-amp/kan (Stratagene, La Jolla, CA, USA). Subsequently, the K-Ras^G12V^-IRES-GFP cassette was cloned via *Not*I digestion into the pTET-bsr vector [22], yielding pTET/K-Ras^G12V^-IRES-GFP-bsr. To achieve doxycycline-inducible expression of oncogenic K-Ras, Caco-2tet cells, stably expressing the doxycycline-inducible system components rtTA and rtTS [21], were transfected with the *Ahd*I-linearized pTET/K-Ras^G12V^-IRES-GFP-bsr vector by electroporation. Following selection with blasticidine S (5 µg/ml) and puromycin (5 µg/ml), resistant cell pools were screened for efficient induction of K-Ras^G12V^ expression. Transgene expression was induced by addition of 2 µg/ml doxycycline (Merck).

### FACS analysis

Analysis of transgene expression of the Caco-2tet cells was performed after 72 dox treatment. Cells were washed, resuspended in PBS containing 2% FCS and 0.01% sodium azide, and analyzed using an EPICS FC500 (Beckman Coulter, Krefeld, Germany). Post-acquisition data analysis was performed using FlowJo software (Tree Star; Ashland, OR, USA).

### Western blotting

Cells were lysed in RIPA buffer (50 mM Tris (pH 7.5), 150 mM NaCl, 1% Triton-X 100, 0.5 sodium deoxycholate, 0.1% SDS, 1 mM sodium orthovanadate, 10 mM sodium fluoride and 20 mM β-glycerophosphate plus Complete protease inhibitors (Roche)). For 3D lysates, cells were cultured on pure matrigel without collagen. Spheroids were isolated after 4 days using Cell Recovery Solution (BD) and lysed in RIPA buffer. Lysates were clarified by centrifugation, equal amounts of protein were separated by SDS–PAGE (NuPAGE Novex Bis-Tris Gel; Invitrogen) and transferred to nitrocellulose membrane (iBlot Gel Transfer Stacks; Invitrogen). Membranes were blocked with 0.5% blocking reagent (Roche) in PBS containing 0.1% Tween-20 and incubated with primary antibodies, followed by HRP-conjugated secondary antibodies. Visualization was done with ECL detection system (Pierce, Rockford, IL, USA).

### MTT, cytotoxicity, and caspase 3/7 activity assays

For 2D cultures, 2.5×10^3^ cells/well in 100 µl medium were plated into uncoated 96-well plates. For 3D cultures, 5×10^3^ cells/well were seeded into matrigel/collagen-coated 96-well plates in 100 µl medium containing 2% matrigel. Viability was determined by addition of 10 µl 3-(4,5-dimethylthiazol-2yl-)2,5-diphenyl tetrazolium (MTT; Roth, Karlsruhe, Germany) solution (5 mg/ml) followed by incubation for 3 h. Cells were lysed by addition of 100 µl 50% dimethylformamide containing 10% SDS and absorbance was measured at 570 nm using the multimode reader Infinite 200 PRO (Tecan, Männedorf, Switzerland). Cytotoxicity was measured using the CytoTox-Glo Cytotoxicity Assay from Promega (Madison, WI, USA). The activity of dead-cell protease in the culture was determined by addition of 50 µl luminogenic substrate. After 15 min incubation at RT, luminescence was measured using the multimode reader Infinite 200 PRO (Tecan), followed by cell lysis and measurement of total luminescence for normalization.

Caspase 3/7 activity was determined using the Caspase-Glo3/7 Assay from Promega (Madison, WI, USA) by addition of 70 µl luminogenic substrate containing the DEVD sequence. After 30 min incubation at RT, luminescence was measured using the multimode reader Infinite 200 PRO (Tecan).

### Tunel staining

DNA strand breaks were analyzed with the in situ cell death detection kit (TMR) from Roche. Cells were fixed with 4% PFA for 1 h at RT and permeabilized with 0.1% Triton-X 100 in 0.1% sodium citrate for 2 min at RT. Labeling was performed according to the manufacturer’s protocol for 1 h at 37°C. Nuclei were counterstained with DAPI. Slides were mounted in Fluoromount G (Southern Biotechnology, Birmingham, AL, USA) and analyzed on a confocal laser scanning microscope (LSM 700; Zeiss, Oberkochen, Germany). Images were processed with the ZEN software (Zeiss). Tunel-positive cells were counted using ImageJ (W. Rasband, National Institute of Health, USA; Version 1.48).

### Immunofluorescence microscopy

Cells grown in 3D on matrigel/collagen coated 8-well glass chamber slides (BD) were fixed with 4% PFA for 15 min, permeabilized with PBS containing 0.1% Triton X-100 for 10 min and blocked with 5% goat serum (Invitrogen) in PBS containing 0.1% Tween-20. Cells were then incubated with primary antibodies in blocking buffer (2 h at RT), washed with PBS containing 0.1% Tween-20 and incubated with secondary antibody in blocking buffer (2 h at RT). F-Actin and nuclei were counterstained with Alexa Fluor 633-labeled phalloidin and DAPI. Slides were mounted in Fluoromount G and analyzed on a confocal laser scanning microscope (LSM 700; Zeiss, Oberkochen, Germany) using 488, 561 and 633 nm excitation with oil objective lenses Plan-Apochromat 63x/1.40 DIC M27. Images were processed with the ZEN software (Zeiss).

### Statistical analysis

Data are expressed as mean (± S.E.M.), and ‘n’ refers to the number of independent experiments. Statistical significance was evaluated by t-test, and one-way ANOVA followed by Tukey’s post-test (GraphPad Prism version 4.03; GraphPad Software Inc., La Jolla, CA). p-values below 0.05 were considered significant (*p<0.05; **p<0.01; ***p<0.001; ns, p>0.05).

## Results

**Figure 1:**
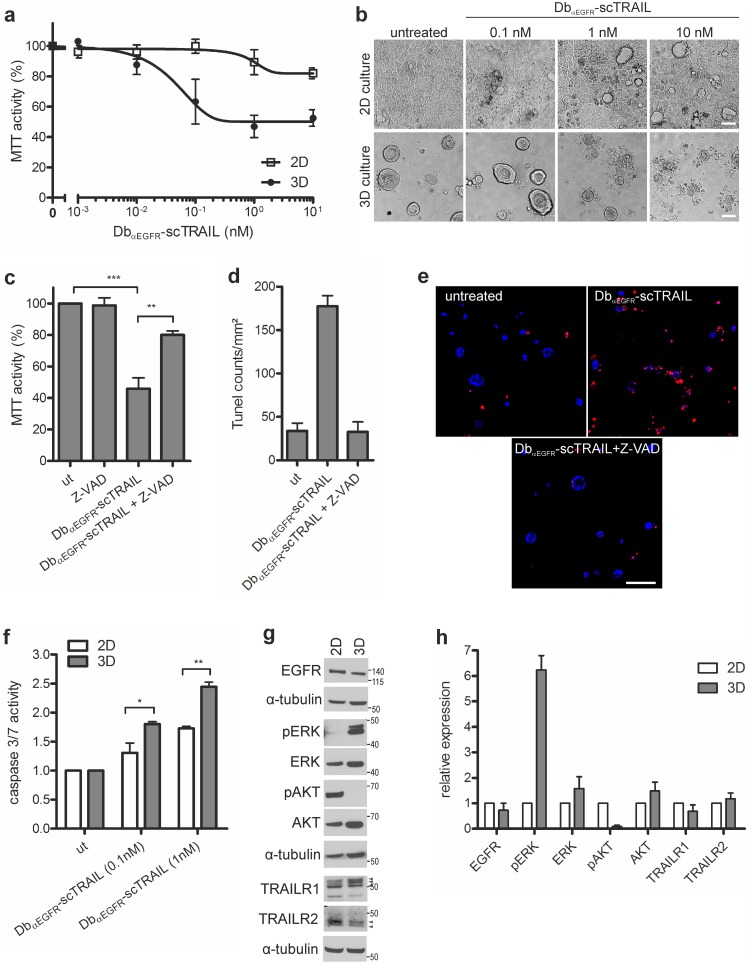
Caco-2 3D cultures are sensitive to Db_αEGFR_-scTRAIL. Cells were grown in 3D or 2D in medium containing 10% FCS. (a) Three days post seeding, cultures were treated with Db_αEGFR_-scTRAIL. Viability was measured 72 h later by MTT assay and normalized to the untreated control (n = 3). (b) Phase contrast images of the 3D and 2D cultures described in (a) treated with the indicated concentrations of Db_αEGFR_-scTRAIL for 72 h (scale bar: 50 µm). (c) Three days post seeding, 3D cultures were pretreated with 20 µM Z-VAD as indicated before addition of 1 nM Db_αEGFR_-scTRAIL. Viability was measured 72 h later by MTT assay and normalized to the untreated control (ut) (n = 3). (d) 24 h after treatment, cells were fixed and stained for DNA strand breaks. Tunel-positive cells were counted (n = 2). (e) Representative pictures of the Tunel stainings described in (d), Tunel-positive cells (red), DAPI (nuclei; blue). Shown are confocal sections (scale bar: 100 µm). (f) Three days post seeding, cultures were treated with 0.1 nM or 1 nM Db_αEGFR_-scTRAIL for 24 h. Caspase 3/7 activity was measured and normalized to the respective untreated control (ut) (n = 3). (g) Four days post seeding, lysates were generated and analyzed by immunoblotting. Shown is one representative blot of three independent experiments. Tubulin was detected as a loading control. Specific bands are marked by arrowheads. (h) Quantification of Western blots from (g). Protein levels were normalized to the corresponding tubulin control; levels in the 2D cultures were set as 1 (n = 3).

In 3D matrigel cultures, Caco-2 cells differentiate into polarized cysts composed of a single cell layer surrounding a central lumen [17], [21], reflecting the organotypic organization of the colon. Because these cells are EGFR-positive and express wild-type Ras, they represent an ideal model system for studying the combined effect of EGFR inhibition and an apoptosis-inducing agent such as TRAIL. To first test the efficacy of Db_αEGFR_-scTRAIL, Caco-2 cells were cultured for three days in 3D in medium containing 10% FCS before addition of Db_αEGFR_-scTRAIL followed by MTT measurements three days later. In these cultures, relatively low doses of Db_αEGFR_-scTRAIL caused a significant reduction of cell viability which was associated with the disruption of cysts and the formation of apoptotic bodies (Fig. 1a, b), scTRAIL alone or in combination with Cetuximab failed to elicit a cytotoxic response in Caco-2 3D cultures (Fig. S1), supporting our previous data that the diabody-mediated dimeric structure of Db_αEGFR_-scTRAIL confers superior bioactivity over scTRAIL [15]. Interestingly, in conventional 2D cell cultures on plastic, Caco-2 cells were highly resistant to Db_αEGFR_-scTRAIL treatment (Fig. 1a, b), in line with a previous report using recombinant human TRAIL [23]. Pretreatment of the Caco-2 3D cultures with Z-VAD, a pan-caspase inhibitor, significantly reduced the cytotoxic effect of Db_αEGFR_-scTRAIL (Fig. 1c), and the induction of apoptosis by Db_αEGFR_-scTRAIL was confirmed by the analysis of DNA fragmentation by Tunel staining (Fig. 1d, e). Additionally, compared with 2D cultures, the dose-dependent activation of caspases 3/7 in response to Db_αEGFR_-scTRAIL was significantly increased in 3D cultures (Fig. 1f). This difference in sensitivity toward Db_αEGFR_-scTRAIL in 3D versus 2D cultures could not be attributed to changes in EGFR or TRAILR1/2 expression (Fig. 1g, h). Unfortunately, because the decoy receptors DcR1, DcR2 could not be detected by immunoblotting with the antibodies available, expression changes in these receptors could not be ruled out. Analysis of key signaling pathways revealed that, in 3D cultures, the activity of the PI3K pathway was suppressed compared with cells grown in 2D as measured by phospho-Akt levels whereas the ERK/MAPK pathway was upregulated as seen by increased ERK1/2 phosphorylation (Fig. 1g, h). However, inhibition of PI3K by LY294002 in 2D cultures was not sufficient to sensitize cells to Db_αEGFR_-scTRAIL (data not shown), indicating a more complex scenario in 3D cultures. Together, these results underscore the impact of the culture conditions on the cellular response toward apoptosis-inducing agents.

**Figure 2:**
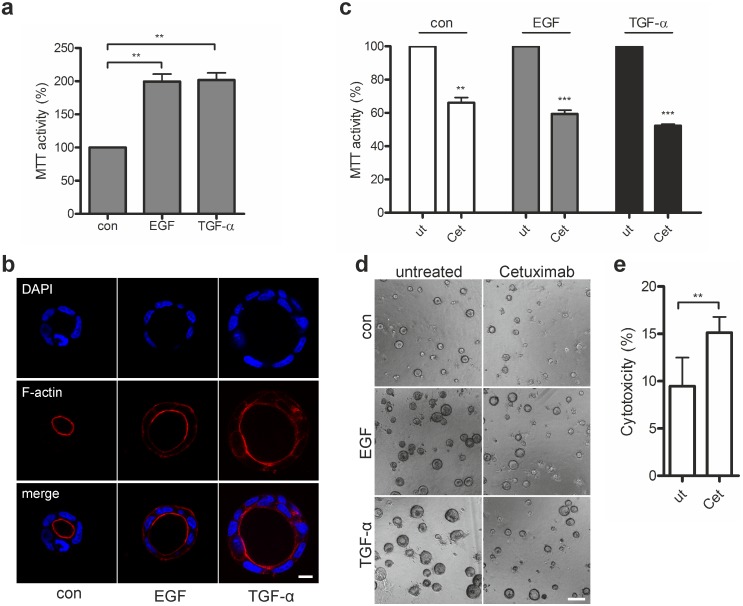
Caco-2 3D cultures are sensitive to pharmacological EGFR inhibition. Caco-2 cells were grown in 3D cultures containing 2% FCS in the presence of growth factors (10 ng/ml) or in 2% FCS only (con). (a) Cultures were analyzed by MTT assay at day 6 and normalized to the control (n = 5). (b) Three days post seeding 100 ng/ml CTX was added to induce lumen expansion. Spheroids were fixed on day 6 and stained with phalloidin (F-actin) and DAPI (nuclei). Shown are confocal sections of a representative cyst (scale bar: 10 µm). (c) Three days after seeding, 3D cultures were left untreated or treated with 0.5 µM Cetuximab (Cet) for 72 h. Viability was determined by MTT assay and normalized to the untreated control (ut). (n = 4) (d) Caco-2 3D cultures were left untreated or treated with 0.5 µM Cetuximab for 72 h and analyzed by phase microscopy (scale bar: 50 µm). (e) Three days after seeding, 3D cultures were left untreated (ut) or treated with 0.5 µM Cetuximab (Cet) for 72 h. Cytotoxicity was determined using the CytoTox-Glo Cytotoxicity Assay (n = 3).

We next investigated how the presence of EGFR ligands affected growth and differentiation of Caco-2 cells in the 3D cultures. Cells were seeded in matrigel cultures containing low serum (2%) in the presence of EGF or TGF-α, ensuring that proliferation was mainly driven via EGFR signaling. MTT activity measurements after six days of cultivation indicated that EGF and TGF-α enhanced the proliferation of Caco-2 cells compared with control cells grown in low serum only (Fig. 2a). Microscopic analysis revealed that in the presence of EGF and TGF-α Caco-2 cysts were larger and contained more cells (Fig. 2b). Notably, the addition of EGFR ligands did not interfere with differentiation, as judged by the typical apical distribution of F-actin and the formation of a cell-free lumen (Fig. 2b). To address how EGFR blockade affected basal and EGFR ligand-induced proliferation of established Caco-2 cysts, we treated the cells three days after seeding with Cetuximab (0.5 µM) and analyzed the cultures three days later. Compared with the control, in these cultures the MTT activity was significantly reduced by 35–50%, but cells were still viable, did not show any signs of apoptosis and only negligibly increased cytotoxicity (Fig. 2c–e; S1d). This indicates that EGFR activation contributes to basal proliferation, but is not required for survival. Cetuximab also potently inhibited proliferation in the presence of EGF and TGF-α as seen by the reduction of MTT activity and the reduced size of the cysts (Fig. 2c, d). Together these experiments demonstrate that proliferation of Caco-2 cells in 3D cultures can be driven by EGFR signaling and is sensitive to pharmacological EGFR inhibition, and can thus potentially be suppressed by Db_αEGFR_-scTRAIL.

**Figure 3:**
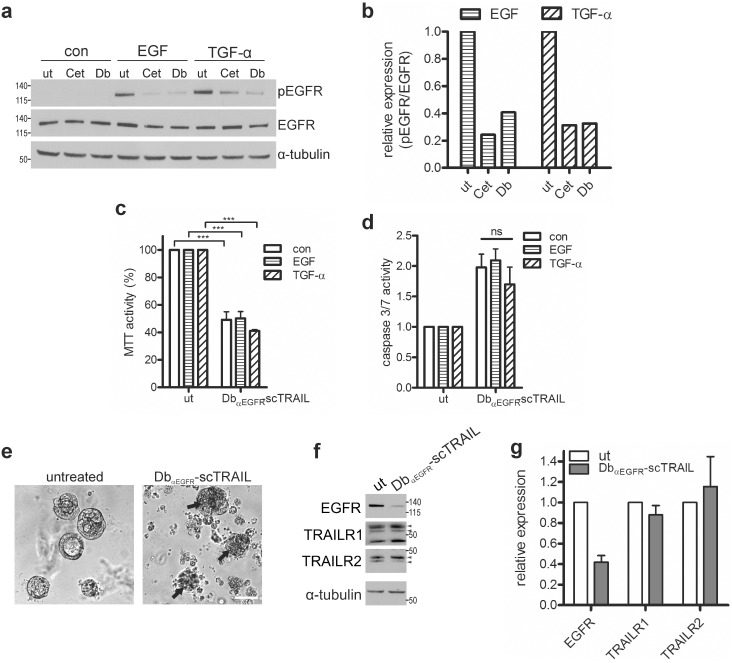
EGFR-directed targeting determines the bioactivity of Db_αEGFR_-scTRAIL. (a) Caco-2 cells grown in 2D were left untreated or treated with 4 nM Db_αEGFR_-scTRAIL or 4 nM Cetuximab for 15 min prior to stimulation with EGF or TGF-α (10 ng/ml) for 10 min. Phosphorylated and total proteins were detected by immunoblotting. Shown is one representative blot of three independent experiments. Tubulin was detected as a loading control. (b) Quantification of Western blots from (a). Phospho-EGFR levels were normalized to the corresponding total protein levels; levels in the untreated control were set as 1 (n = 3). (c, d). Three days post seeding, Caco-2 3D cultures grown in the absence or presence of growth factors were treated with 1 nM Db_αEGFR_-scTRAIL. (c) Viability was determined by MTT assay after 72 h and normalized to the respective untreated control (ut). (n = 3) (d) Caspase 3/7 activity was measured after 24 h. Values were normalized to the corresponding untreated control (n = 3). (e) Three days post seeding, Caco-2 3D cultures were either left untreated or treated with 5 nM Db_αEGFR_-scTRAIL for 72 h. Surviving cysts in the phase contrast images are indicated by arrows (scale bar: 50 µm). (f) Lysates derived from the 3D cultures shown in (e) were analyzed by immunoblotting. Shown is one representative blot of three independent experiments. Tubulin was detected as a loading control. Specific bands are marked by arrowheads. (g) Quantification of Western blots from (f). Protein levels were normalized to the corresponding tubulin control; levels in the untreated cultures were set as 1 (n = 3).

EGFR activation not only stimulates cell proliferation but can also protect from TRAIL-induced apoptosis [24]. Therefore, we next explored the efficacy of Db_αEGFR_-scTRAIL in the presence of EGFR ligands. Immunoblotting of lysates of EGF- and TGF-α-stimulated cells revealed a similar degree of suppression of ligand-induced EGFR phosphorylation by either Cetuximab or Db_αEGFR_-scTRAIL pretreatment (Fig. 3a, b), indicating the efficient competition of the diabody moiety with the EGFR ligands. This could also be confirmed in 3D cultured Caco-2 cells stimulated with EGF (Fig. S2). Accordingly, EGF and TGF-α had no protective effect on viability in 3D (Fig. 3c) nor were these ligands able to interfere with caspase activation (Fig. 3d), demonstrating that Db_αEGFR_-scTRAIL activity is not limited by the presence of EGFR ligands. To understand in more detail resistance mechanisms toward Db_αEGFR_-scTRAIL, we re-isolated cysts from untreated and Db_αEGFR_-scTRAIL-treated 3D matrigel cultures followed by immunoblotting of cell lysates (Fig. 3f, g). Interestingly, lysates derived from surviving cysts (Fig. 3e, arrows) revealed that these Db_αEGFR_-scTRAIL-insensitive cells contained especially low EGFR levels. Of note, TRAIL receptor levels in these cells were similar to those in untreated cells, suggesting that the distribution of EGFR expression in the cell population strongly impacts Db_αEGFR_-scTRAIL sensitivity (Fig. 3f, g). Thus, although the diabody moiety does not actively contribute to apoptosis and mainly has a growth inhibitory function in Caco-2 3D cultures, the EGFR-directed targeting is important to increase the local TRAIL concentration and trigger an efficient apoptotic response.

**Figure 4:**
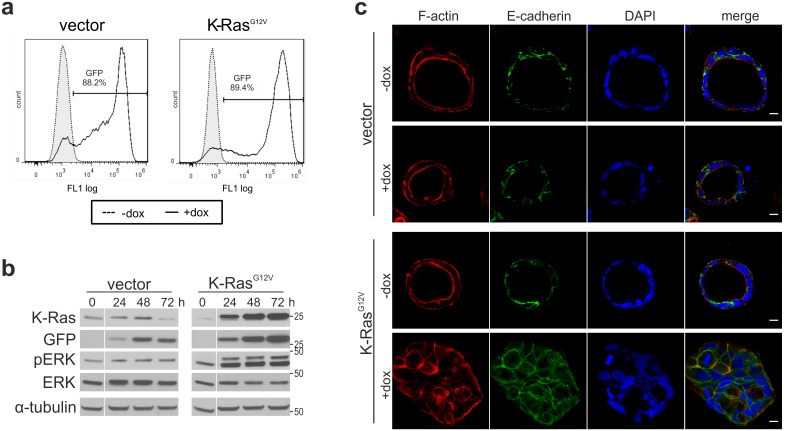
Inducible expression of oncogenic K-Ras^G12V^ in Caco-2tet cells. (a) Caco-2tet vector control and K-Ras^G12V^ cells were treated with 2 µg/ml doxycycline for 72 h (+dox). Cells were harvested and GFP fluorescence was analyzed by flow cytometry. Non-induced cells were used as a control (−dox). (b) Caco-2tet vector control and K-Ras^G12V^ cells were grown in 2D and treated with doxycycline for the indicated times prior to lysis. GFP, Ras, pERK (T202/Y204) and ERK levels were determined by Western blotting. Tubulin was detected as a loading control. All panels shown are from the same blot. (c) Caco-2tet vector control and K-Ras^G12V^ cells were seeded into 3D cultures in the absence or presence of doxycycline. Three days post seeding lumen expansion was induced by addition of 100 ng/ml CTX. Cultures were fixed three days later and stained with E-cadherin-specific antibody (green), phalloidin (red) and DAPI (nuclei; blue). Shown are confocal sections of representative cysts (scale bar: 10 µm).

Approximately 40% of all CRC tumors harbor an active mutation in the *KRAS* gene, leading to constitutive ERK/MAPK activation and loss of responsiveness to Cetuximab [7], whereas TRAIL sensitivity may be increased [25]. To investigate the influence of oncogenic Ras on Db_αEGFR_-scTRAIL-induced cytotoxicity, we generated stable Caco-2 cells inducibly expressing K-Ras^G12V^. In these cells, doxycycline induces the bi-cistronic expression of the oncogene and GFP, whereas vector control cells express GFP only. Three days after doxycycline addition, more than 85% of the Caco-2tet cells were GFP positive by FACS analysis (Fig. 4a). Immunoblotting of Caco-2tet K-Ras^G12V^ cell lysates confirmed Ras overexpression along with that of GFP, concomitant with strong ERK phosphorylation, whereas vector control cells expressed only GFP (Fig. 4b). When these cells were seeded into 3D cultures in the absence of doxycycline, both Caco-2tet vector and K-Ras^G12V^ cells formed well-differentiated and polarized spheroids with basolateral adherens junctions (E-cadherin staining) and apical F-actin accumulation around a cell-free lumen. Addition of doxycycline had no effect on the morphology of the control cells, whereas the K-Ras^G12V^ expressing cells formed multi-luminal spheroids that lacked distinct polarization (Fig. 4c). These differentiation defects caused by K-Ras^G12V^ are in accordance with a recent report by Magudia et al. (2012) [16].

**Figure 5:**
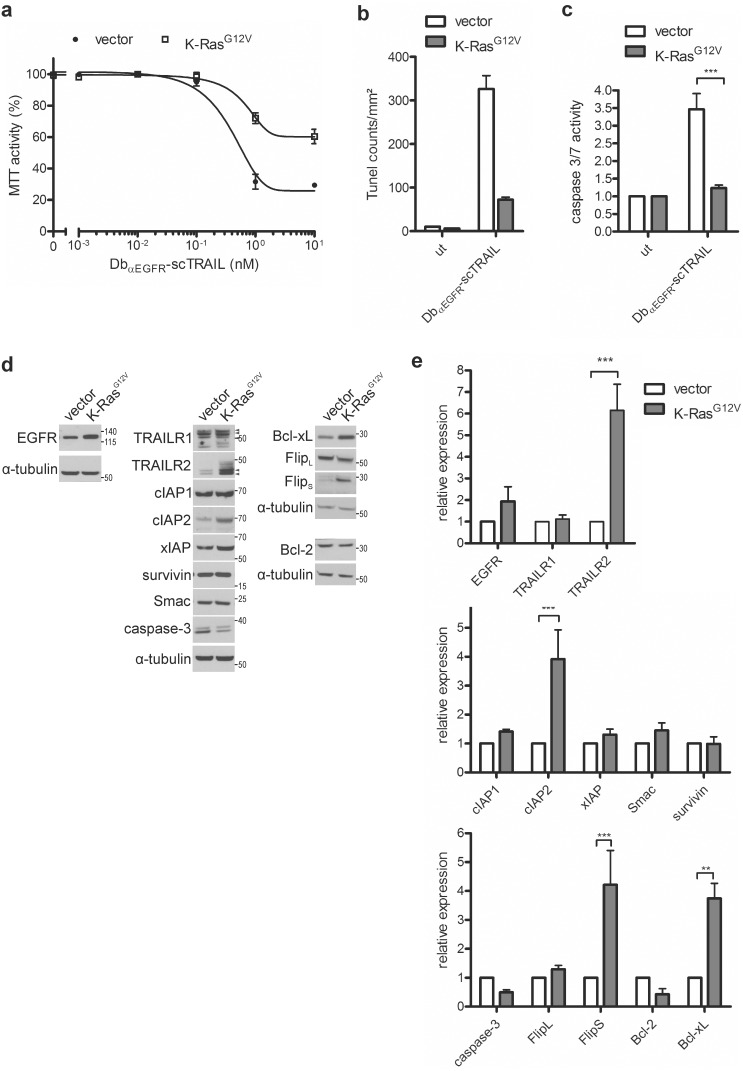
Oncogenic K-Ras protects Caco-2tet cells from Db_αEGFR_-scTRAIL induced apoptosis. (a–c) Caco-2tet vector control and K-Ras^G12V^ cells were seeded into 3D cultures in the presence of doxycycline. Three days later, cultures were treated with Db_αEGFR_-scTRAIL. (a) Viability was measured 72 h later by MTT assay and normalized to the untreated control (n = 3). (b) 24 h after treatment with 1 nM Db_αEGFR_-scTRAIL, cells were fixed and stained for DNA strand breaks. Tunel-positive cells were counted (n = 2). (c) Caspase 3/7 activity was measured after 24 h treatment with 1 nM Db_αEGFR_-scTRAIL. Values shown were normalized to the corresponding untreated control (ut) (n = 3). (d) Caco-2tet vector control and K-Ras^G12V^ cells were grown in 3D cultures in the presence of doxycycline for 4 days. Cells were recovered from the 3D cultures and lysates were analyzed by immunoblotting. Shown is one representative blot of three independent experiments. Tubulin was detected as a loading control. Specific bands are marked by arrowheads. (e) Quantification of Western blots from (c). Protein levels were normalized to the corresponding tubulin control; levels in the vector control were set as 1 (n = 3).

To determine the effects of K-Ras^G12V^ expression on Db_αEGFR_-scTRAIL induced cytotoxicity, 3D cultures were treated with doxycycline for three days. Compared with the control cells, viability measurements revealed decreased sensitivity of oncogenic Ras expressing cells to Db_αEGFR_-scTRAIL (Fig. 5a). To confirm that this partial resistance was due to reduced apoptosis, we performed Tunel stainings and measured caspase 3/7 activation. Indeed, K-Ras^G12V^ cells showed strongly reduced DNA fragmentation after treatment with 1 nM Db_αEGFR_-scTRAIL (Fig. 5b) and only a weak induction of caspase activity (Fig. 5c). This resistance was not due to EGFR or TRAILR1/2 downregulation. In fact, immunoblotting of 3D cell lysates of the vector and K-Ras^G12V^ cells revealed increased TRAILR2 protein levels (Fig. 5d, e), in agreement with previous reports [25], [26]. Oncogenic Ras can interfere with apoptosis at multiple levels, for example, by activation of PI3K survival signaling and changes in transcriptional programs [27], [28]. Indeed, in 2D cultures, we observed increased Akt phosphorylation in K-Ras^G12V^ expressing cells. In 3D cultures, however, the suppression of the PI3K-Akt pathway appears to be dominant (see Fig. 1g), with K-Ras^G12V^ expression leading to marginally elevated Akt phosphorylation only (data not shown). Interestingly, expression analysis of selected key regulators of the apoptotic machinery revealed significantly elevated levels of the anti-apoptotic proteins cIAP2, Flip_S_, and Bcl-xL (Fig. 5d, e).

**Figure 6:**
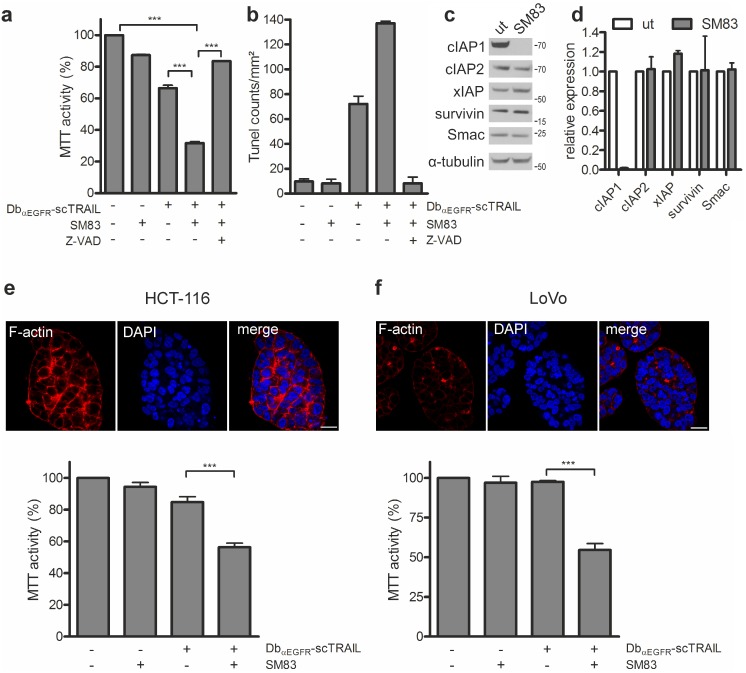
The Smac mimetic SM83 sensitizes oncogenic K-Ras expressing CRC cells to Db_αEGFR_-scTRAIL. (a–d) Caco-2tet Ras^G12V^ cells were seeded into 3D cultures in the presence of doxycycline. (a) Three days later, cultures were left untreated or treated with 5 µM SM83 or 20 µM Z-VAD for 1 h prior to addition of 1 nM Db_αEGFR_-scTRAIL. Viability was measured 24 h later by MTT assay and normalized to the untreated control (n = 3). (b) 24 h after treatment, cells were fixed and stained for DNA strand breaks. Tunel-positive cells were counted (n = 2). (c) Three days post seeding, cultures were left untreated (ut) or treated with 5 µM SM83. Cells were recovered from the cultures 24 h later and lysates were analyzed by immunoblotting. Shown is one representative blot of two independent experiments. Tubulin was detected as a loading control. (d) Quantification of Western blots from (c). Protein levels were normalized to the corresponding tubulin control; levels in the untreated cultures were set as 1 (n = 2). (e, f) HCT-116 and LoVo cells were grown in 3D cultures for six days, and then fixed and stained for F-actin and DNA (DAPI) (scale bar: 20 µm) (top panels). Three days post seeding, cultures were left untreated or pretreated with 5 µM SM83 prior to addition of 0.05 nM Db_αEGFR_-scTRAIL. Viability was measured 24 h later by MTT assay and normalized to the untreated control (bottom panels) (n = 3).

Because molecular changes occurred at different levels of the apoptotic pathway, we sought to block K-Ras^G12V^ induced anti-apoptotic signaling as far downstream as possible. Inhibitor of apoptosis (IAP) proteins, such as cIAP2, interfere with apoptosis by the direct binding, inhibition and/or degradation of caspases and components of the Ripoptosome, and by antagonizing non-canonical NFkappaB signaling [29]. The activity of IAP proteins is balanced by Smac/Diablo, a protein released from mitochondria in cells primed for apoptosis. Peptides that mimic the aminoterminal IAP-binding sequence of Smac, so-called Smac mimetics, were found to enhance the cytotoxicity of chemotherapeutic agents and death ligands such as TRAIL [30], [31]. Indeed, co-treatment of Caco-2tet K-Ras^G12V^ cells with Db_αEGFR_-scTRAIL and a previously developed highly efficient dimeric Smac mimetic, SM83 [19], [20], decreased cell viability by 35% compared with the single treatments. This was efficiently blocked by Z-VAD, proving the involvement of caspase activation in the case of the combinatorial treatment (Fig. 6a). Tunel staining further confirmed the enhancement of apoptosis by SM83 (Fig. 6b). Of note, the presence of SM83 also lowered the Db_αEGFR_-scTRAIL concentration required to kill parental Caco-2 and Caco-2tet vector cells (data not shown). Analysis of cell lysates derived from Caco-2 3D cultures showed that SM83 incubation for 24 h led to the complete loss of cIAP1, whereas cIAP2 levels were only slightly decreased, and xIAP, survivin and Smac were not affected (Fig. 6c, d). The presence of cIAP2 at this time point can be explained by its upregulation in response to cIAP1 loss in accordance with a previous report (Fig. S3; [32]). Nevertheless, by regulating the overall IAP/Smac balance, SM83 appears to restore the apoptotic response in oncogenic Ras expressing Caco-2 cells. Finally, to investigate whether our targeted combination strategy could be transferred to CRC cell lines with endogenous *KRAS* mutations, we co-treated HCT-116 and LoVo cells with Db_αEGFR_-scTRAIL and SM83 in 3D cultures. These cell lines are EGFR-positive (data not shown) and show moderate sensitivity to Db_αEGFR_-scTRAIL, making them amenable to the combined action of Db_αEGFR_-scTRAIL and Smac mimetics. Reminiscent of the Caco-2tet K-Ras^G12V^ cells, HCT-116 and LoVo cells failed to form differentiated and polarized spheroids in 3D culture (Fig. 6e, f). Importantly, a synergistic cytotoxic effect of Db_αEGFR_-scTRAIL and SM83 was observed for both cell lines when Db_αEGFR_-scTRAIL was applied at a sublethal concentration (Fig. 6e, f). Thus, based on our data we propose that EGFR-targeted scTRAIL molecules, together with apoptosis-sensitizing agents, may be an effective therapy for CRC independently of the *KRAS* status.

## Discussion

Inducing apoptosis exclusively in tumor cells, while leaving normal tissue unharmed, is the goal of every anti-cancer drug development. Due to its selectivity for tumor cells, TRAIL is regarded as a promising anti-cancer therapeutic. Apoptosis induction by TRAIL moreover does not depend on p53, the frequent loss or mutation of which is a major cause of chemotherapy resistance [33]. Despite these advantageous features of TRAIL, the molecular mechanisms that govern TRAIL sensitivity versus resistance still remain poorly understood. There is neither a clear correlation between total TRAILR1/2 levels nor the ratio of TRAILR1/2 and the decoy receptors DcR1, DcR2 and TRAIL sensitivity [34], [35]; instead, activation of PI3K/Akt and NFkappaB signaling pathways, and the overexpression of anti-apoptotic proteins such as Bcl-2, IAPs and Flip have been implicated in TRAIL resistance [36], [37], [9]. A better understanding of resistance mechanisms and the identification of effective drug combinations are thus essential for the right choice of target patients and an optimized personalized treatment design.

Here, we explored in a 3D model of CRC the mechanisms of action of a targeted single-chain TRAIL molecule comprising an EGFR blocking and targeting moiety derived from Cetuximab and three TRAIL monomers. EGFR ligands have been reported to protect epithelial cells from TRAIL-induced apoptosis, mainly via the activation of PI3K signaling [24], [38]. Treatment of CRC cells with recombinant TRAIL was further reported to lead to EGFR upregulation and shedding of TGF-α, resulting in the activation of autocrine and paracrine EGFR/HER2 pro-survival signaling [39]. Additionally, EGFR ligand shedding may also be induced by oncogenic Ras signaling [40]. These findings provide a rationale for combining pharmacological EGFR blockade with TRAIL receptor agonists. Indeed, in an ErbB2/Neu tumor mouse model the combined treatment with an ErbB2 blocking antibody and a TRAILR2 agonistic antibody had synergistic effects [41]. Consistent with this, our data show that Db_αEGFR_-scTRAIL reduced basal and EGFR ligand-induced proliferation of Caco-2 cells in 3D cultures in addition to efficiently inducing apoptosis of EGFR-positive cells irrespective of the presence of the EGFR ligands EGF or TGF-α.

Using Caco-2tet cells inducibly expressing K-Ras^G12V^, we provide evidence that oncogenic Ras protects from death receptor-induced apoptosis. In CRC, ligand-independent activation of the ERK/MAPK and PI3K pathways by mutant K-Ras is not only associated with the loss of responsiveness to Cetuximab [7], but also enhances cell proliferation and survival by interfering with the apoptotic machinery. Specifically, *KRAS* mutations at codon 12 could be correlated to reduced apoptosis in vitro and lower apoptotic indices in colorectal tumors [42]. By contrast, K-Ras has also been reported to promote apoptosis through the upregulation of TRAILR2 [25], [28]. Intriguingly, in doxycycline-treated Caco-2tet Ras^G12V^ cells TRAILR2 was also found to be upregulated, however, this was not sufficient to trigger apoptosis. It appears that the additional molecular changes in the apoptotic pathway downstream of TRAIL receptors, such as the elevation of Flip_S_, Bcl-xL and cIAP2 overcome TRAILR2 upregulation, ultimately favoring cell survival. The balance of pro- and anti-apoptotic signaling molecules thus appears to determine the cellular outcome in a cell type-dependent manner.

This balance between proliferation, survival and death is not only dictated by cell-intrinsic factors, but is also profoundly affected by the cellular environment. Our data reveal that Caco-2 cells display dramatically increased sensitivity to Db_αEGFR_-scTRAIL-induced apoptosis in 3D collagen/matrigel cultures, which cannot simply be explained by changes in EGFR or TRAIL1/2 receptor levels. Because clustering is important for the stimulation of TRAIL receptor activation, the basolateral localization of both EGFR and TRAIL receptors may create a more densely packed signaling platform that facilitates death receptor oligomerization [43], [44]. In addition, the polarized growth in the presence of extracellular matrix components will alter the activation states of intracellular signaling pathways (Fig. 1g, h). The suppression of the PI3K pathway in Caco-2 3D cultures is likely to contribute to the enhanced TRAIL sensitivity, as activation of this pathway correlated with TRAIL resistance in several tumor cell lines and PI3K inhibition rendered cells more sensitive toward TRAIL treatment [12], [45], [46]. Regardless of the precise mechanism, the impact of the cell culture set-up should be considered in any studies involving in vitro drug testing.

Expression analyses revealed that oncogenic Ras induced the strong upregulation of cIAP2 in Caco-2tet cells. Alterations in IAPs are found in many types of human cancer and associated with chemoresistance, disease progression and poor prognosis [47], [29]. In intestinal epithelial cells, Ras was shown to cause cIAP2 upregulation via a TGF-α autocrine loop [48]. Our data are consistent with this observation and support the potential benefit of blocking autocrine EGFR signaling in combination with death receptor stimulation. cIAP proteins bind, but do not inhibit caspases 3 and 7, promoting their ubiquitination and degradation [49]. This explains the reduced caspase 3 levels observed in Caco-2tet Ras^G12V^ cells, although this downregulation was not significant. Smac mimetics were originally developed to block xIAP, but they are most effective at triggering the auto-ubiquitination and degradation of cIAP1 and cIAP2. In our 3D CRC model, strongly reduced cIAP1 levels were detected 24 hours after SM83 treatment, whereas cIAP2 was only transiently downregulated, in accordance with the finding that cIAP1 downregulation causes cIAP2 upregulation by non-canonical NFkappaB activation [32]. Therefore, the potent downregulation of cIAP1 by SM83 appears to be sufficient to tip the balance and restore an apoptotic response to Db_αEGFR_-scTRAIL. Smac mimetics have further been reported to induce cytotoxicity as single agents, a feature linked to the induction TNF-α synthesis and secretion [50], [51]. However, in Caco-2, HCT-116 and LoVo cells, we did not observe any cytotoxicity in response to SM83 alone, suggesting that the increased susceptibility to death receptor-induced apoptosis primarily stems from TRAIL receptor signaling.

Finally, a very important aspect uncovered by our study is the requirement for tumor cell-specific targeting of recombinant TRAIL to elicit a potent cytotoxic response. Intriguingly, cells surviving Db_αEGFR_-scTRAIL treatment in the 3D cultures expressed very low EGFR levels. It can thus be assumed that patients whose tumors express high EGFR levels should respond best to Db_αEGFR_-scTRAIL. Directing recombinant single-chain TRAIL molecules to tumor-specific surface antigens using a diabody-based forced dimerization strategy is not limited to the EGFR and together with appropriate apoptosis sensitizing agents may be a powerful approach to efficiently kill a broad range of cancer cells.

## Supporting Information

Figure S1 **Db_αEGFR_-scTRAIL bioactivity is superior to scTRAIL.** Caco-2 cells were grown in 3D cultures in medium containing 10% FCS. (a, b) Three days post seeding, cultures were left untreated (ut) or treated with 1 nM Db_αEGFR_-scTRAIL or scTRAIL. (a) Cell viability was determined by MTT assay after 72 h and normalized to the untreated control (n = 3). (b) Caspase 3/7 activity was measured after 24 h. The values shown were normalized to the untreated control (n = 3). (c, d) Three days post seeding, cultures were left untreated (ut) or treated with 1 nM scTRAIL, 1 nM Cetuximab or the combination of both. (c) Cell viability was determined by MTT assay after 72 h and normalized to the untreated control (n = 3). (d) Caspase 3/7 activity was measured after 24 h. The values shown were normalized to the untreated control (n = 3).
(TIF)

Figure S2 **Db_αEGFR_-scTRAIL potently inhibits EGFR activation.** (a) Caco-2 cells grown in 3D for 3 days were left untreated or treated with 4 nM Db_αEGFR_-scTRAIL or 4 nM Cetuximab for 15 min prior to stimulation with EGF (10 ng/ml) for 10 min. Phosphorylated and total proteins were detected by immunoblotting. Tubulin was detected as a loading control. (b) Quantification of Western blots from (a). Shown is the ratio of phosphorylated EGFR to total EGFR; levels in the untreated control were set as 1 (n = 2).
(TIF)

Figure S3 **Downregulation of cIAP1 and cIAP2 by SM83.** Caco-2tet Ras^G12V^ cells grown in 2D for 72 h in the presence of dox followed by treatment with 5 µM SM83 for the indicated time points prior to lysis. Proteins were analyzed by immunoblotting using the indicated antibodies. Tubulin was detected as a loading control.
(TIF)
