# Supplementary figures and images for: EGFR-Targeted TRAIL and a Smac Mimetic Synergize to Overcome Apoptosis Resistance in KRAS Mutant Colorectal Cancer Cells
Source: PLoS One. 2014 Sep 8;9(9):e107165. doi: 10.1371/journal.pone.0107165 (PMC4157814; doi:10.1371/journal.pone.0107165)

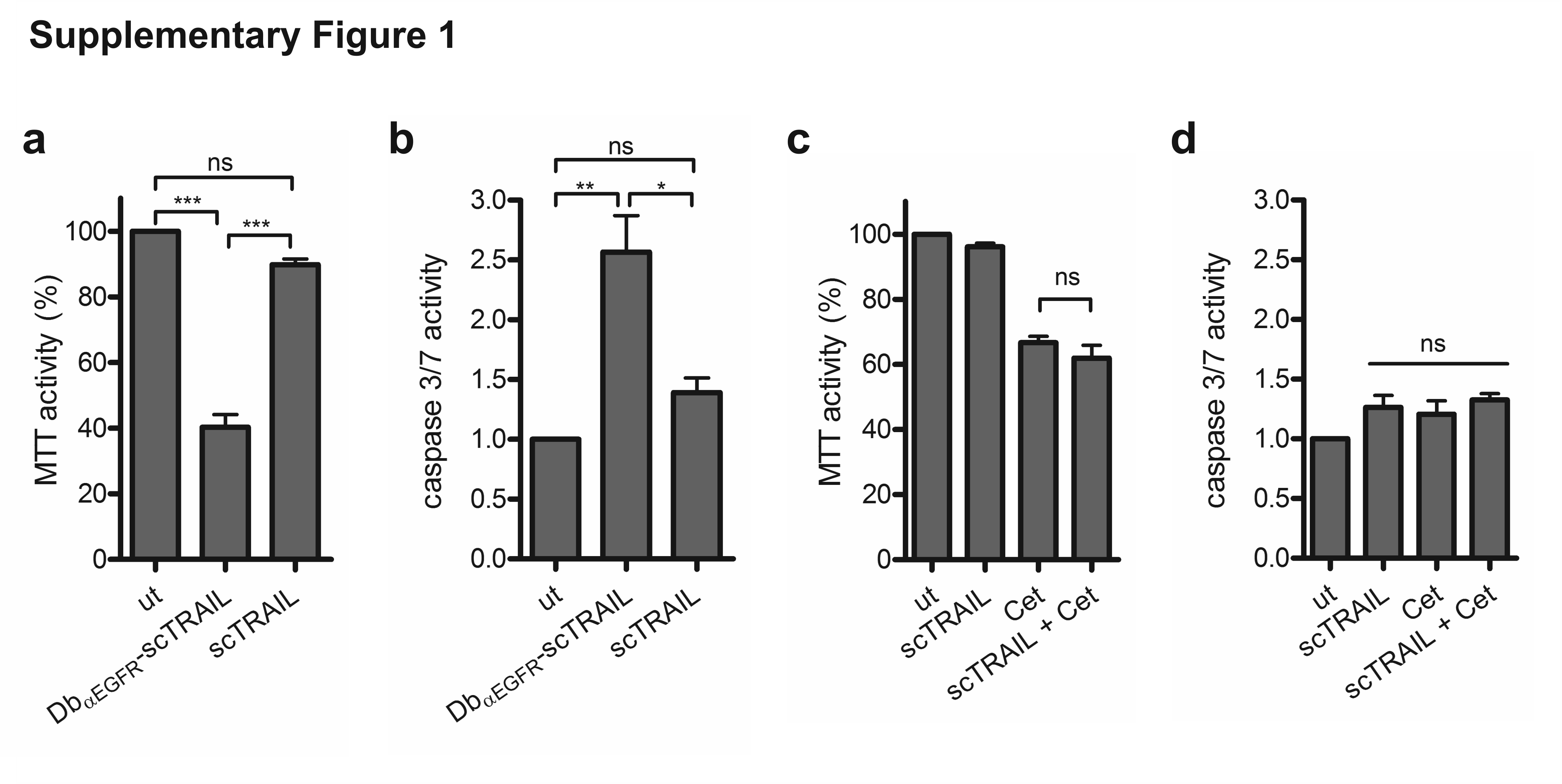

Supplement: Figure S1 — DbαEGFR-scTRAIL bioactivity is superior to scTRAIL. Caco-2 cells were grown in 3D cultures in medium containing 10% FCS. (a, b) Three days post seeding, cultures were left untreated (ut) or treated with 1 nM DbαEGFR-scTRAIL or scTRAIL. (a) Cell viability was determined by MTT assay after 72 h and normalized to the untreated control (n = 3). (b) Caspase 3/7 activity was measured after 24 h. The values shown were normalized to the untreated control (n = 3). (c, d) Three days post seeding, cultures were left untreated (ut) or treated with 1 nM scTRAIL, 1 nM Cetuximab or the combination of both. (c) Cell viability was determined by MTT assay after 72 h and normalized to the untreated control (n = 3). (d) Caspase 3/7 activity was measured after 24 h. The values shown were normalized to the untreated control (n = 3). (TIF) [file pone.0107165.s001.tif]

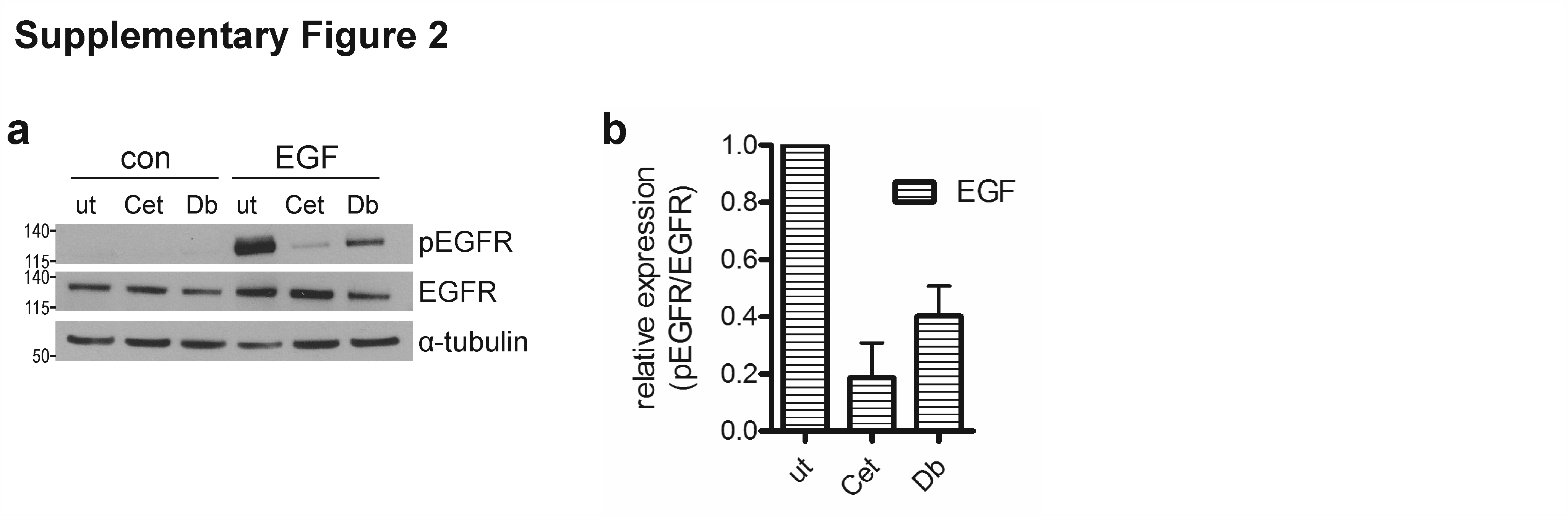

Supplement: Figure S2 — DbαEGFR-scTRAIL potently inhibits EGFR activation. (a) Caco-2 cells grown in 3D for 3 days were left untreated or treated with 4 nM DbαEGFR-scTRAIL or 4 nM Cetuximab for 15 min prior to stimulation with EGF (10 ng/ml) for 10 min. Phosphorylated and total proteins were detected by immunoblotting. Tubulin was detected as a loading control. (b) Quantification of Western blots from (a). Shown is the ratio of phosphorylated EGFR to total EGFR; levels in the untreated control were set as 1 (n = 2). (TIF) [file pone.0107165.s002.tif]

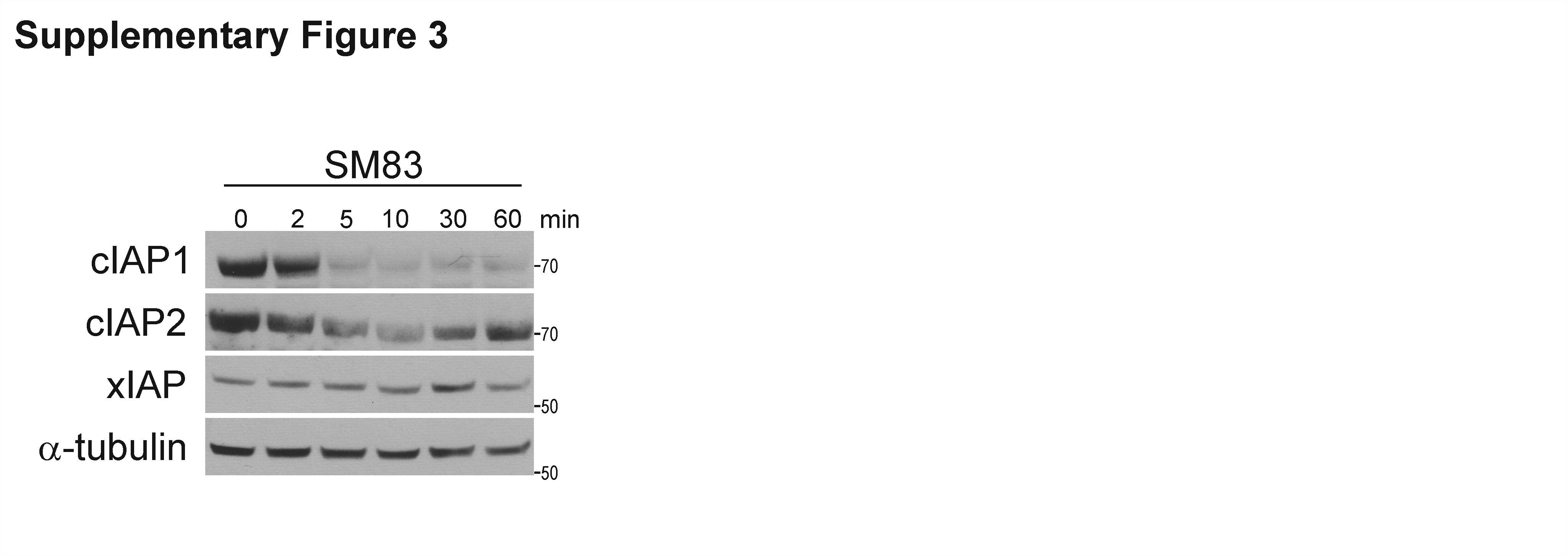

Supplement: Figure S3 — Downregulation of cIAP1 and cIAP2 by SM83. Caco-2tet RasG12V cells grown in 2D for 72 h in the presence of dox followed by treatment with 5 µM SM83 for the indicated time points prior to lysis. Proteins were analyzed by immunoblotting using the indicated antibodies. Tubulin was detected as a loading control. (TIF) [file pone.0107165.s003.tif]
